# The Central Role and Possible Mechanisms of Bacterial DNAs in Sepsis Development

**DOI:** 10.1155/2020/7418342

**Published:** 2020-08-31

**Authors:** Zhenxing Cheng, Simon T. Abrams, James Austin, Julien Toh, Susan Siyu Wang, Zhi Wang, Qian Yu, Weiping Yu, Cheng Hock Toh, Guozheng Wang

**Affiliations:** ^1^Department of Clinical Infection, Microbiology and Immunology, Institute of Infection and Global Health, The University of Liverpool, Liverpool, L69 7BE, UK; ^2^Medical School, Southeast University, Nanjing 21009, China; ^3^Wirral University Teaching Hospitals NHS Foundation Trust, Arrowe Park Road, Upton, Wirral CH49 5PE, UK; ^4^Royal London Hospital, Whitechapel Rd, Whitechapel, London E1 1FR, UK; ^5^Liverpool University Hospitals NHS Foundation Trust, Liverpool L7 8XP, UK

## Abstract

The pathological roles of bacterial DNA have been documented many decades ago. Bacterial DNAs are different from mammalian DNAs; the latter are heavily methylated. Mammalian cells have sensors such as TLR-9 to sense the DNAs with nonmethylated CpGs and distinguish them from host DNAs with methylated CpGs. Further investigation has identified many other types of DNA sensors distributed in a variety of cellular compartments. These sensors not only sense foreign DNAs, including bacterial and viral DNAs, but also sense damaged DNAs from the host cells. The major downstream signalling pathways includeTLR-9-MyD88-IKKa-IRF-7/NF-*κ*B pathways to increase IFN/proinflammatory cytokine production, STING-TBK1-IRF3 pathway to increase IFN-beta, and AIM2-ASC-caspas-1 pathway to release IL-1beta. The major outcome is to activate host immune response by inducing cytokine production. In this review, we focus on the roles and potential mechanisms of DNA sensors and downstream pathways in sepsis. Although bacterial DNAs play important roles in sepsis development, bacterial DNAs alone are unable to cause severe disease nor lead to death. Priming animals with bacterial DNAs facilitate other pathological factors, such as LPS and other virulent factors, to induce severe disease and lethality. We also discuss compartmental distribution of DNA sensors and pathological significance as well as the transport of extracellular DNAs into cells. Understanding the roles of DNA sensors and signal pathways will pave the way for novel therapeutic strategies in many diseases, particularly in sepsis.

## 1. Introduction

Damage-associated molecular patterns (DMAPs), including extracellular histones and DNAs, neutrophil extracellular traps (NETs) and other factors, mediate multiple organ injury and play key roles in critical illnesses, particularly in sepsis [1–3]. Extensive immune cell death often occurs in sepsis and becomes a major source of DMAPs [4, 5], but the underlying molecular mechanism is not clear. We found that bacterial DNAs play a major role in priming immune system and lead to extensive death of immune cells if a second hit appears. However, this is a neglected field because many reports on DNA sensors and signalling pathways mainly focus on cancer and viral infection [6–8]. Here, we will review the available literatures of DNA sensors and signalling pathways that are related to bacterial infection and sepsis.

During bacterial infections, bacterial DNA is released after bacterial breakdown and enters the circulation. The DNA in circulation can then be rapidly cleared by the spleen and the liver with a half-life (*t*½) of approximately 4 minutes [9, 10]. However, if the bacterial infection cannot be controlled, large quantities of bacterial DNA could continuously enter the circulation and trigger severe immune responses [11–13]. Circulating DNA taken up by the spleen stimulates lymphocytes to release cytokines [14]. High levels of circulating bacterial DNAs have a direct association with the metrics of clinical severity, suggesting that bacterial DNA levels are highly reflective of bacterial load [15]. Bacterial DNA-triggered immune responses play important roles in the elimination of human pathogens [16–18].

## 2. Types of DNAs in Inflammation

CpG motifs in bacterial DNAs trigger B cell activation [11]. In eukaryotic DNA, CpG motifs have a high degree of methylation, which may allow the host to distinguish them from the bacterial DNA [19], which is largely unmethylated. Methylation of CpG residues blocks their capacity of stimulating immune responses [20]. Bacterial DNA has been demonstrated to stimulate natural killer (NK) cells to increase lytic activities and interferon *γ* (IFN-*γ*) production, stimulate lymphocytes to secrete interleukin 6 and 12 (IL-6, IL-12) as well as IFN-*γ*, stimulate macrophages to produce tumour necrosis factor *α* (TNF-*α*) [11, 12, 21–24], and activate neutrophils [25]. Elevated TNF-*α* promotes the development of shock, and elevated IFN-*γ* primes macrophage and natural killer (NK) cells to increase the toxicity of lipopolysaccharides (LPS) [23, 24, 26–28]. In the presence of DNA-binding proteins, such as HMGB1, which is a CpG-DNA-binding protein, B lymphocyte (B cells) activation is significantly enhanced either by bacterial DNA [29] or by a synthesized 6-base nucleotide motif of an unmethylated CpG dinucleotide flanked by 2 purines (5′) and 2 pyrimidines (3′). This nucleotide motif is present 20 times more in bacterial DNA than in vertebrate DNA [11, 30, 31]. The sequence specificity was demonstrated in direct DNA immunization using plasmid DNA carrying the necessary sequences for immune activation [32–34].

## 3. Receptors and Signal Pathways

Mammalian cells express a variety of DNA sensors as one of the first lines of defence against infection [35, 36]. TLR-9 is a major receptor that recognises bacterial DNA and signals in host cells [37]. The TLR family are pattern recognition receptors activated by diverse conserved components of pathogens called pathogen-associated molecular patterns (PAMPs). In vertebrates, strong selective pressure maintains the highly conservative TLR receptors' recognition of and response to PAMPs [38]. TLR-9 receptors are expressed in many tissues including the spleen, where it is expressed most abundantly, and in many types of immune cells, including macrophages, lymphocytes, dendritic cells, natural killer cells, neutrophils, and other antigen-presenting cells [39]. UNC93B1 [40–42], PRAT4A [43, 44], and adaptor protein AP3 [45] transport TLR-9 from the endoplasmic reticulum to the endosome or the lysosome, where TLR-9 is matured after cleavage of its ectodomain by cathepsins [41, 46, 47]. Specific innate immune cells take up extracellular bacterial materials into endosomes and phagosomes, where the N-terminal domain of TLR-9 senses the unmethylated CpG DNA motifs [48, 49]. HMGB1 also forms complexes with TLR-9, and this causes translocation of TLR-9 to endosomes in response to CpG-DNA and CpG-oligodeoxynucleotides (CpG-ODN), which ultimately triggers cytokine responses [29]. CpG-DNA activates TLR-9 to generate signalling through myeloid differentiation primary response gene 88 (MyD88), which in turn enhance IFN expression and activate proinflammatory and antimicrobial signals via mitogen-activated protein kinase (MAPK) and/or nuclear factor *κ*B (NF-*κ*B) pathways [50, 51]. However, different cell types may vary in downstream pathways, for instance, in plasmacytoid DCs, myddosome forms to activate IRF-7 and increase IFN*α* production [52]. In contrast, macrophages rarely produce type-1 IFN in response to TLR9 ligands unless using DOTAP as the transfection reagent to deliver CpG-DNA into the cells [53]. However, TLR-9 and TLR-4 crosstalk amplifies macrophage responses through activation of the c-Jun N-terminal kinases (JNKs) [54]. In lymphocytes, ligand-inducible high expression of TLR-9 was found in activated B lymphocytes [55]. DNA-containing antigen-triggered B cell responses rely on a TLR-9-dependent immune checkpoint [56].

Cyclic GMP-AMP synthase (cGAS), a cytosolic DNA sensor [57–59] also senses CpG DNA. However, cGAS recognises cytosolic double-stranded DNA in a sequence-independent manner and cannot distinguish microbial DNA from host DNA. cGAS-binding DNA produces cyclic GMP-AMP (cGAMP) to activate STING and signal via TBK1 and IFN regulatory factor (IRF)-3 to induce expression of type I IFN [60–62]. This signal also activates the NF-*κ*B pathway to enhance inflammatory cytokine release, including TNF-*α* and IL-6, which mediate inflammatory and antimicrobial responses [63–68]. STING also activates stress and cell death pathways that lead to T cell [69] and B cell death [70]. In chicken cells, DEAD box polypeptide 41 (DDX41) is an important DNA sensor to induce IFN production, which also depends on the STING pathway [71]. These observations strongly suggest that signalling through the STING pathway is a very important response to both cytosolic viral and bacterial DNA and mediates IFN-dependent innate immunity [72]. A STING-independent DNA sensing pathway (SIDSP) has also been identified. For example, DNA damage response protein DNA-PK was reported recently as the primary sensor in the SIDSP, which mainly activates the pathway via HSPA8/HSC70 in humans but is absent from mouse cells [73].

**Figure 1:**
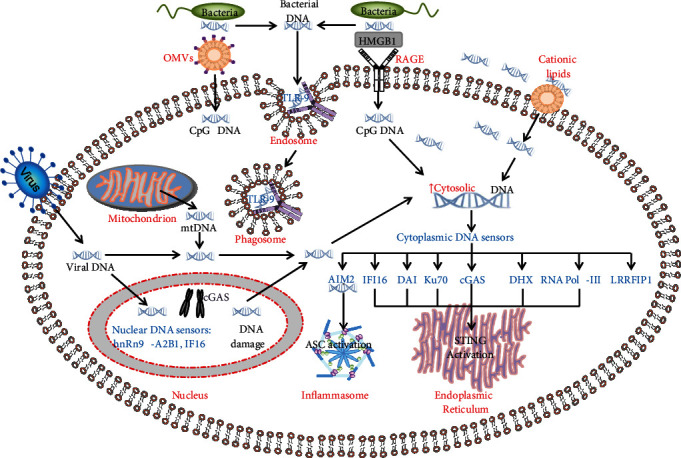
Compartmental distribution of DNA sensors and internalization of external DNAs. TLR-9 is the most important sensor for bacterial DNAs and is located on the membrane of endosomes and phagosomes. AIM2 binds to cytosol DNAs, which initiate the assembly of inflammasomes by activating ASC. Many other cytosolic DNA sensors have been identified, and most of them activate STING pathways, including cGAS, IFI16, DAI, Ku70, DHX9, DHX36, DHX41, and RNA Pol-III. LRRFIP1 is also a cytosolic DNA sensor but it mainly triggers the beta-catenin pathway. cGAS and IFI16 also exist in cell nuclei but cGAS binds to chromosome to enhance its stability whist nuclear cGAS and IFI16 plus hnRNP-A2B1-DNA can detect viral DNAs which enter nuclei and form complexes with DNAs. The complexes are exported to the cytosol to activate STING pathways. Most DNA sensors are situated inside cells, and the extracellular DNAs from virus, bacteria, or host cells enter cells by many different ways, including endocytosis and phagocytosis, or are facilitated by HMGB1-RAGE, cationic lipids, or OMVs. Viruses enter cells via different membrane proteins.

Absent in melanoma 2 (AIM2), a protein with a N-terminal pyrin domain and a C-terminal HIN-200 domain, is an important component of the inflammasome [74, 75]. AIM2 senses cytoplasmic DNA to activate ASC (apoptosis-associated speck like protein) pyroprotosomes and caspase-1 to ultimately cause cell death [76–81]. LL37 peptide can neutralize cytosolic DNA and block AIM2-mediated inflammasome activation [82]. IFI16 is another member of the HIN family and binds viral DNA to regulate IL-1*β* maturation, but does not associate with ASC [83]. IFI16 is predominantly a nuclear protein which translocates to the cytosol to recognize DNA and induces IFN-*β* production. IFI16 also forms complexes with viral DNA in the nucleus, and these complexes translocate to the cytosol to trigger STING-dependent signalling [83–88]. Many other DNA sensors have been reported, including DNA-dependent activator of IFN-regulatory factor (DAI, also called Z-DNA binding protein-1, ZBP1) [89], leucine-rich repeat interacting protein-1 (Lrrfip1) [90], RNA polymerase III [91, 92], Ku70, and DExD/H box helicases (DHX9 and DHX36) [93]. DAI recognizes both the B-form and Z-form of cytosolic ds-DNA. LRRFIP1 induces IFN production via a *β*-catenin-dependent pathway [90], whilst RNA polymerase III transcribes DNA into 5′-ppp RNA to activate RIG-1-STING pathway, which triggers IFN production [91, 92]. Ku70 acts as a cytosolic DNA sensor to promote type III IFN-*λ*1 production via activation of IRF1and IRF7 [94]. DHX36 detects CpG-A DNA using the DEAH domain to mediate IFN-*α* production via the MyD88-IRF7 pathway, whilst DHX9 senses CpG-B DNA using the DUF domain to increase TNF-*α* and IL-6 production via the MyD88-NF-*κ*B pathway [93, 95, 96]. Recently, heterogeneous nuclear ribonucleoprotein A2/B1 (hnRNP-A2B1) has been shown to recognise viral double-stranded DNA inside the cell nucleus but not the host DNA that is packed as nucleosomes. The hnRNP-A2B1-DNA complexes translocated into the cytosol induce IFN production via STING-dependent pathways [97]. The major pathways initiated by DNA sensors are summarized in Figure 1.

## 4. Compartmental Distribution of DNA Sensors

As mentioned above, each DNA sensor is located in a specific compartment (see Figure 1). TLR-9 exists in endosomes and phagosomes to detect DNAs that entered the cell via endocytosis or phagocytosis [53]. There are many cytosolic DNA sensors, including DAI, Ku70, RNA pol III, DHX9/36, and LRRFIP1, to detect cytosolic DNA. AIM2 is located in the inflammasome, hnRNP-A2B1 in nucleus, and IFI16 in the nuclear plasma. cGAS attached to the plasma membrane by N-terminal phosphoinositide-binding domain detects viral DNAs, whilst mutant cGAS in the cytosol mainly responds to host stress instead of viral DNAs [98]. In contrast, chromatin-bound cGAS inhibits DNA repair to increase genome stability and promotes cell death [99], indicating the importance of compartmentalisation of DNA sensor proteins.

DNA sensors distributed in a variety of cell compartments may play an important role in sensing bacterial and viral DNA that enter cells via different mechanisms so as to ensure swift recognition by the host. However, T and B cells, unlike macrophages, do not take up DNA which limits their response to bacterial DNAs, particularly high molecular weight DNA [100]. When DNA is physically linked to hen egg lysozyme (HEL), the resulting complex was efficiently taken up by B cells in the experimental setting [100]. In bacterial infections, besides endocytosis, many other factors, such as HMGB1-RAGE [101], cationic lipids [102], and outer membrane vesicles (OMVs) [103], may facilitate bacterial DNA uptake by host cells and trigger DNA sensors and downstream signal pathways.

## 5. STING Plays a Central Role in Both Exogenous and Endogenous DNA Sensing

**Figure 2:**
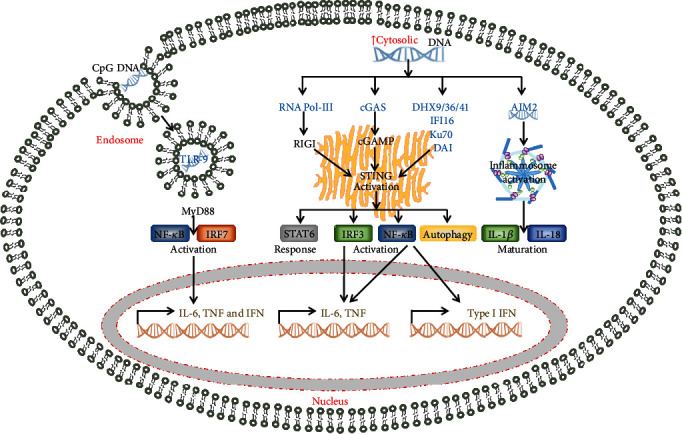
Major signalling pathways of DNA sensors. TLR-9-MyD88, STING, and AIM2-ASC are the major pathways initiated by DNA sensors. TLR9 preferentially binds bacterial and viral DNAs to trigger NF-*κ*B and IRF7 signaling cascades via MyD88 adaptor and lead to a proinflammatory cytokine response. STING is a common pathway of many DNA sensors to induce IFN and other proinflammatory cytokines. AIM2-ASC induces inflammasome assembly and activation to process IL-1beta and IL-18.

DNA-sensing receptor cyclic GMP–AMP synthase (cGAS) activating the STING pathway plays a central role in sensing and responding to cytosolic DNA from both exogenous and endogenous sources. Endogenous nuclear DNA damage caused by ionizing radiation, oxidative stress, drugs, telomere shortening, chromosome mis-segregation, mitochondrial damage, and viral/bacterial infection all lead to accumulation of DNA in the cytosol [104]. Once the DNA is exposed to the cGAS sensor, cGAS forms dimers and synthesizes cyclic guanosine monophosphate-adenosine monophosphate (cGAMP). The cGAMP acts as a second messenger to activate STING on the ER surface, which ultimately activates IRF3 and NF-*κ*B through the kinases TBK1 and IKK, respectively, so as to increase expression of the cytokines including IFN and TNF-*α* [73, 104, 105] (see Figure 2). On the other hand, STING can also mediate IFI16 degradation by reducing type I interferon production [106]. Many other factors, such as cyclic di-nucleotides from intracellular pathogens, DHX41, RNA Pol-III, DAI, or IFI16, also activate STING pathway (Figure 1) and initiate different responses mediated by IRF3, NF-*κ*B, STAT6 [107], and autophagy [108] pathways (Figure 2). Therefore, the cGAS-STING pathway is an important regulator of free DNA resulting from infection, inflammation, or cancer [104, 109, 110].

Activation of the STING pathway not only causes cytokine release, but also induces apoptosis in immune cells, including in T and B lymphocytes [111], myeloid lineage cells, and in nonimmune cells, such as hepatocytes [112, 113] and cardiomyocytes [114], and in cancer cells [70, 110, 115]. STING pathway-induced apoptosis involves ER stress and Bak/Bax-mediated macropores in the mitochondrial outer membrane or leakage of lysosomal content into the cytosol [69, 114, 116]. Recent reports demonstrate that STING signalling plays an important role in the induction of necrosis via the synergistic effect between IFN and TNF-*α* pathways [118; 119].

## 6. Syngeneic Effects with Other Toxic Factors

Although bacterial CpG DNA triggers an immune response via DNA sensors, incubation of cells with the bacterial DNA or injection of DNA into mice is not sufficient to cause cell or animal death [117]. CpG DNA motifs were reported as sensitizing agents for lipopolysaccharide-induced toxic shock in animal models [117]. CpG-DNA and LPS can act synergistically to induce inflammatory cytokines and nitric oxide release, and to increase cell-surface DNA binding and internalisation in monocytes and macrophages [118–122]. CpG DNA and LPS synergistically induce TNF-*α* production via activation of NF-*κ*B [120, 123]. High doses of D-galactosamine is lethal in animal models [124], whilst lower doses (nonlethal) are used to sensitize animals [125]. In sensitized mice, CpG DNA or LPS is able to induce acute liver injury via the mitochondrial apoptotic pathway involving TLR pattern recognition receptors [126–128]. Galactosamine-induced sensitization also contributes to endotoxin-induced immune response and lethality [129, 130].

## 7. Roles of Bacterial DNAs in Disease

Bacterial infection causes variety of diseases with different mechanisms and manifestations. The majority of bacteria do not enter host cells, but releases DNAs which are transported into cells via endocytosis or phagocytosis to meet TLR-9 receptor [37]. Some bacteria could enter host cells and release DNAs directly into cytosol to trigger cytosolic DNA sensors [131].

It was reported that bacterial DNA could cause septic shock via induction of high levels of TNF-*α* [132]. However, no confirmatory report has since been published. In general, bacterial DNA causes a variety of immune responses, but no lethal effects, although blocking TLR-9 reduces bacterial load, inflammation, and mortality in mouse poly-microbial sepsis [133]. Kukoamine B, a novel dual inhibitor of LPS and CpG-DNA, has been reported to be effective in treating animals with sepsis [134]. Intratracheal admission of synthetic CpG-ODNs leads to acute lung inflammation and injury with systemic inflammatory response via activation of TLR-9 [135, 136]. Bacterial DNA induces pulmonary damage via TLR-9, and suppressing CpG-ODNs and TLR-9 could significantly reduce inflammation in the mouse lungs [137, 138]. The same effect has also been reported in cardiac injury and malfunction caused by bacterial DNA [10].

On the other hand, synthetic oligonucleotides have also been used as modulators of inflammation and immune response [139]. Single-stranded DNA containing CpG motifs have also been shown to induce innate immune responses, including production of poly-reactive immunoglobulin and the production of T helper 1 (TH1)-type as well as release of proinflammatory cytokines and chemokines [140] and to activate B lymphocytes [11]. The innate immune responses are able to increase host resistance to a wide range of pathogenic bacteria, viruses, and parasites, and therefore, CpG-ODNs have been tried with vaccines to enhance immunity [141]. When mice are exposed to bacterial DNA before hemorrhagic shock, the systemic inflammation and gut barrier loss via an IFN-*γ*-dependent route are strongly aggravated [142]. CpG ODNs has also been shown to stimulate protective innate immunity, enhance the complement system, protect immune cells, increase secretion of antibacterial antibody, and enhance phagocytosis against intracerebral *E. coli* K1 infection, pulmonary Klebsiella, or *Staphylococcus aureus* infections [143–148]. These observations indicate that there are both harmful and beneficial effects of bacterial DNA.

## 8. Potential Diagnostic and Therapeutic Strategy and Reagents

DNase, particularly DNase 1, has been used directly in many animal models to digest extracellular DNAs, including DNAs in dead bacteria and neutrophil extracellular traps (NETs) [149–151]. It is very likely that DNase 1 will be used clinically in the near future [152]. The other strategy is to target the sensors of bacterial DNAs and downstream pathways, mainly the TLR-9 and cGAS-STING pathways. As mentioned above, targeting TLR-9 improves the outcomes of mouse sepsis [133]. Targeting the TLR-9-MyD88 pathway is also used to regulate adaptive immune responses [153], but the majority of studies were focused on cancer [154]. Many drugs targeting TLR-9 and other TLRs are under clinical trials, mainly for cancer therapy [153, 155]. However, targeting TLRs has demonstrated a great potential in sepsis management and infection control, as well as optimisation of vaccine efficacy [156, 157]. STING-knockout mice developed less severe acute pancreatitis than wild type mice, whilst STING agonists cause more severe acute pancreatitis [158]. However, the STING ligand, c-di-GMP, can improve vaccination results for metastatic breast cancer [159]. Recently, the STING pathway has become much more attractive in targeting both inflammation and cancer [160]. STING agonists also reduce the burden of pancreatic cancer in mouse models [161]. As such, many STING pathway agonists and antagonists have been developed [162–164]. Although the science behind this pathway is becoming clearer, successful development of medication targeting this pathway appears a long way off.

Since the major outcome of DNA priming is the production of IFN-*γ* and TNF-*α*, which are the key cytokines of the generalized Shwartzman reaction [27], reports have been accumulated that demonstrate the roles of IFN-*γ* and its receptors in animal models of sepsis and endotoxemia. IFN-*γ*-sensitized mice are more susceptible to LPS-induced mortality [23, 27, 165]. Thus, IFN-*γ*-/- mice showed a significant reduction in infection-induced splenic cell apoptosis [166]. Moreover, IFN-*γ* receptor-deficient mice gain resistance to LPS-induced septic shock [167]. Anti-IFN-*γ* has also been demonstrated to have protective effects in animal models [168, 169]. However, no successful clinical trials on sepsis have been reported yet, although some clinical trials on the roles of anti-IFN-*γ* in sepsis-induced immune dysfunction are nearing completion. There is no evidence to demonstrate whether blocking the pathway of IFN-*γ* production, such as using DNase 1, would be a better choice.

## 9. Conclusion

The roles of different DNAs and sensors are still a major area of scientific research. How these sensors and pathways are coordinated to function in different types of cells still needs further clarification. Moreover, the cross-talk between the inflammatory pathways and the cell proliferation or death pathways has been shown to be very important in elucidating the pathological mechanisms in the development of diseases. In sepsis, there is a big gap between bacterial DNA priming and immune cell death and subsequently disease development, although it is known that the priming process is very important. Thus, more work needs to be done both in vitro and in vivo to elucidate the signalling pathways and to identify the targets. Sepsis is a common disease with an unacceptable mortality rate, but no specific therapy is yet available. Clarification of the roles and molecular mechanisms of bacterial DNAs in development of sepsis may lead to the creation of new therapies to increase the survival of patients with sepsis.
